# Co-expression Profiling of Autism Genes in the Mouse Brain

**DOI:** 10.1371/journal.pcbi.1003128

**Published:** 2013-07-25

**Authors:** Idan Menashe, Pascal Grange, Eric C. Larsen, Sharmila Banerjee-Basu, Partha P. Mitra

**Affiliations:** 1 MindSpec, McLean, Virginia, United States of America; 2 Department of Public Health, Faculty of Health Sciences, Ben-Gurion University of the Negev, Beer Sheva, Israel; 3 Cold Spring Harbor Laboratory, Cold Spring Harbor, New York, United States of America; Allen Institute for Brain Science, United States of America

## Abstract

Autism spectrum disorder (ASD) is one of the most prevalent and highly heritable neurodevelopmental disorders in humans. There is significant evidence that the onset and severity of ASD is governed in part by complex genetic mechanisms affecting the normal development of the brain. To date, a number of genes have been associated with ASD. However, the temporal and spatial co-expression of these genes in the brain remain unclear. To address this issue, we examined the co-expression network of 26 autism genes from AutDB (http://mindspec.org/autdb.html), in the framework of 3,041 genes whose expression energies have the highest correlation between the coronal and sagittal images from the Allen Mouse Brain Atlas database (http://mouse.brain-map.org). These data were derived from *in situ* hybridization experiments conducted on male, 56-day old C57BL/6J mice co-registered to the Allen Reference Atlas, and were used to generate a normalized co-expression matrix indicating the cosine similarity between expression vectors of genes in this database. The network formed by the autism-associated genes showed a higher degree of co-expression connectivity than seen for the other genes in this dataset (Kolmogorov–Smirnov *P* = 5×10^−28^). Using Monte Carlo simulations, we identified two cliques of co-expressed genes that were significantly enriched with autism genes (A Bonferroni corrected *P*<0.05). Genes in both these cliques were significantly over-expressed in the cerebellar cortex (*P* = 1×10^−5^) suggesting possible implication of this brain region in autism. In conclusion, our study provides a detailed profiling of co-expression patterns of autism genes in the mouse brain, and suggests specific brain regions and new candidate genes that could be involved in autism etiology.

## Author Summary

Autism spectrum disorder (ASD) is a complex neurodevelopmental condition associated with many different genes. However, the neuroanatomical and functional properties of these genes in the brain are largely unknown. Here we examined the co-expression network of 26 genes associated with ASD, using data from the Allen Mouse Brain Atlas, which provides a whole-genome, high-resolution map of gene expression pattern in the adult mouse brain. We discovered that autism genes are significantly more co-expressed than expected by chance, suggesting common neuro-functional properties. We then examined the spatial properties of co-expression modules that are highly enriched with autism genes. Consequently, we found that genes in two of these modules are significantly over-expressed in the cerebellar cortex, and particularly in sections that are predominantly populated by granular cells. These findings provide the essential link between gene networks associated with ASD and specific brain regions, and hence lay out a basis for further exploration of the particular neuronal circuits involved in ASD etiology.

## Introduction

Autism spectrum disorder (ASD) is one of the most prevalent and highly heritable neurodevelopmental disorders in humans [1]–[3]. There is strong evidence that the onset and severity of ASD is governed in part by complex molecular mechanisms affecting the normal development of the brain [1], [4]. While no major anatomical pathology have been observed in brains of ASD cases [5], various molecular and neuroimaging studies have linked several brain regions to ASD. For example, Voineagu *et al.* have found differences in gene expression patterns in the cortex of ASD brain [6]. Cortical regions has also been highlighted in neuroimaging studies of autistic brains along the cerebellum and other brain areas [7], [8]. In addition, other studies have pointed to various molecular mechanisms that might be altered in the autistic brain [9]–[11]. In this realm, genes involved in synapse formation and brain circuitry are consistently found to be dysregulated in people with ASD [12]–[14].

Recent genomic advances have led to the discovery of diverse genetic loci linked to ASD, including chromosomal aberrations [15], [16], copy number variations [11], [17], [18] and both common and rare single nucleotide variations (SNVs) [19]–[24]. Consequently, to date, more than 330 candidate genes have been associated with ASD susceptibility [25] and many more are projected to be found. However, despite the plethora of genetic variations associated with ASD, the molecular mechanisms and neuroanatomical structures underlying ASD traits remain largely unclear.

The mouse model system provides a convenient and safe approach to experimentally study neuroanatomical mechanisms and candidate genes for autism susceptibility [26]–[28]. At present, dozens of single gene knockout and transgenic mice models have been used to elucidate neuropathology that might underlie the autism-like behaviors [29]. Despite the obvious genetic and neuroanatomical differences between mouse and human, mouse models are extremely valuable and effectively used in dissecting out the role of specific gene, pathway, neuron subtype, or brain region in a particular abnormal behavior shared by both these mammals. In this realm, the Allen Brain Atlas of the mouse [30]–[33] provides a comprehensive source of genome-wide high-resolution atlas of gene expression throughout the adult mouse brain. In this study, we utilized this database to examine the spatial co-expression characteristics of genes associated with autism susceptibility. Consequently, we identified several co-expression gene networks that are enriched with autism genes highlighting potential candidate genes and brain regions implicated in autism.

## Methods

### Construction of the autism genes dataset (AutRef84)

The autism gene database, AutDB [34], was used to construct a set of 84 genes, AutRef84, strongly implicated in autism pathogenesis [25], [29]. Genes contained in AutDB were classified into four genetic categories: (1) *Rare*: rare submicroscopic copy number variants and single gene disruptions or mutations directly linked to ASD; (2) *Syndromic*: genes implicated in syndromes in which a subset of affected individuals also develop autistic symptoms; (3) *Association*: small risk-conferring candidate genes with common polymorphisms that have been identified from genome-wide association studies in idiopathic ASD cases; and (4) *Functional*: functional candidate genes relevant to ASD that are not covered by any of the other genetic categories. AutRef84 was generated by filtering out functional candidate genes that lack any experimentally derived genetic link to ASD as well as genes that solely belong to the *Association* classification. The resulting dataset consisted of 64 genes classified within the *rare* classification and 20 within the *syndromic* classification (**Supplementary Table S1**).

### Gene expression dataset

We utilized gene expression data available from the Allen Brain Atlas (ABA) of the mouse brain (http://mouse.brain-map.org) which contains voxelized expression profiles for ∼20,000 genes derived from *in situ* hybridization (ISH) experiments conducted on male, 56-day old C57BL/6J mice) [30]–[33]. Gene expression profiles in the ABA were generated for the mouse brains from processed image data for the sagittal sections of a single hemisphere and, for 4,104 genes of high neurobiological interest, coronal and sagittal sections across the whole brain. We focused on genes for which brain-wide data are available. The ISH data were co-registered to the Allen Reference Atlas, which is partitioned into 49,742 cubic voxels of size 200 microns. A gene labeled *g* has a profile of expression energy presented as a function *E(v,g)* over the brain, where *v* is a voxel label. The Allen Mouse Brain Atlas Addiction Database (http://addiction.brainarchitecture.org/) used this expression dataset to create a high quality brain expression dataset, called *A_best_*, which consists of 3,041 genes with the most highly correlated expression energy profiles between the coronal and sagittal sections [35]. This gene-expression dataset was used for the co-expression analysis discussed below.

### Gene co-expression analysis

For the set of 3,041 genes defined above, a gene-by-gene co-expression matrix was computed as follows:
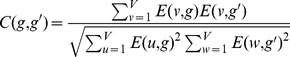
(1)where V = 49,742 is the total number of voxels in the brain. The quantity *C(g,g')* is the cosine similarity between the gene-expression profiles of genes g and g' with values ranging between zero and one by construction. The motivation for choosing the cosine similarity as the co-expression measure in this study, is its connection to the difference between the energies of two brain-wide functions. The proposed co-expression measure (Equation 1) can also be described as a simple decreasing function of the squared energy of the difference between two brain-wide functions. The co-expression matrix *C* is symmetric, with ones on the diagonal. Its size is 3,041, the number of genes in the full *A_best_* dataset. Given a subset of this dataset, the co-expression matrix of this subset can be obtained by extracting the sub-matrix of *C* corresponding to the position of the genes in the full dataset.

To determine whether the autism genes are more co-expressed than other genes expected by chance, the cumulative distribution function (CDF) of the entries of the autism-related and *A_best_* co-expression matrices were compared, and a two-sample Kolmogorov–Smirnov test was used to determine whether they were drawn from the same probability distribution. In addition, we compared the connectivity of the co-expression network of the autism genes to co-expression networks of the same size randomly drawn from *A_best_*. For that, Monte Carlo simulations [36] were conducted to generate 100,000 random gene sets sampled from *A_best_*, and the co-expression matrices of these sets of genes where extracted from the co-expression matrix C.

Given the co-expression matrix of G genes of interest, one can consider the underlying weighted graph with nodes corresponding to genes, and the weight of links equals to the co-expression of the nodes [37]. The matrix can be cut at any value *ρ* between zero and one, resulting in links with weights lower than the threshold *ρ*. At any value of the threshold, the connected components (sets of connected genes) can be computed using Tarjan's algorithm. In particular, the maximum size *M(ρ)* and average size *A(ρ)* of connected components can be calculated for all gene sets over different co-expression values. If *N_ρ_(k)* is the number of connected components in the co-expression matrix of G genes defined by *ρ* threshold and contain exactly k genes out of G genes in the gene set, the maximum and average sizes are expressed as follows:
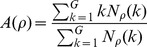
(2)

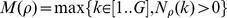
(3)Further, we applied the classical definition of “clique” from graph theory [38], [39] to our co-expression matrix to characterize networks of co-expressed genes such that every gene in the network is connected to all other genes at a co-expression threshold *ρ*.

### Anatomical and functional characterization of co-expression cliques

Next, we aimed at evaluating the unique anatomical properties of co-expression cliques that are significantly enriched with autism genes. For that, we first identified virtually all cliques in the dataset that contained *ng*≥2 autism genes. Then, we used Monte Carlo simulations using 100,000 randomly generated gene sets of size G = 26 to compute the likelihood of each clique to contain at least *ng* autism genes. Finally, for cliques that were significantly enriched with autism genes, we calculated the sum of the normalized expression profiles of the genes in the clique as follow:
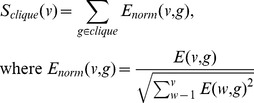
(4)We then used *S_clique_*, to examine the neuroanatomical properties of the genes included in the clique as follows [40]. For a given brain region ω in the Allen Reference Atlas [41], the fitting score between the expression profile and the region ω is defined as the cosine similarity between the expression profile and the characteristic function χ_ω_ of the brain region.
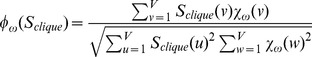
(5)where χ_ω_(v) equals one if voxel labeled v belongs to region ω, and zero otherwise. This fitting score would equal one if the sum of the gene expression in the clique were proportional to the characteristic function of the region ω, and zero if it were entirely supported outside the region. The fitting score defined in the above equation equals the co-expression between a (hypothetical) gene whose expression profile would be *S_clique_*, and a (hypothetical) gene whose expression would coincide exactly with region ω. Fitting scores of a given clique can be computed in all brain regions, and the distribution of these fitting scores can be simulated by repeatedly drawing random cliques of genes (with the same number of genes) from *A_best_*. These analyses were performed using a commercial software package (MATLAB R2011b, The MathWorks Inc., Natick, MA, 2000).

Finally, we used the Bioconductor GOstats package in R software [42] to assess whether genes belonging to a co-expression clique, also share other functional or molecular properties. The absolute list of GO terms were obtained using both a (a) cut-off = 2*ratio (fg/fc) [where fg = frequency of occurrence of a GO term in the given gene set, fc = frequency of its occurrence in the complete list of human genes] and (b) cut-off = median value of ratio (fg/fc). Only significant terms (P<0.01) with an associated gene count> = 5 were considered.

## Results

**Figure 1:**
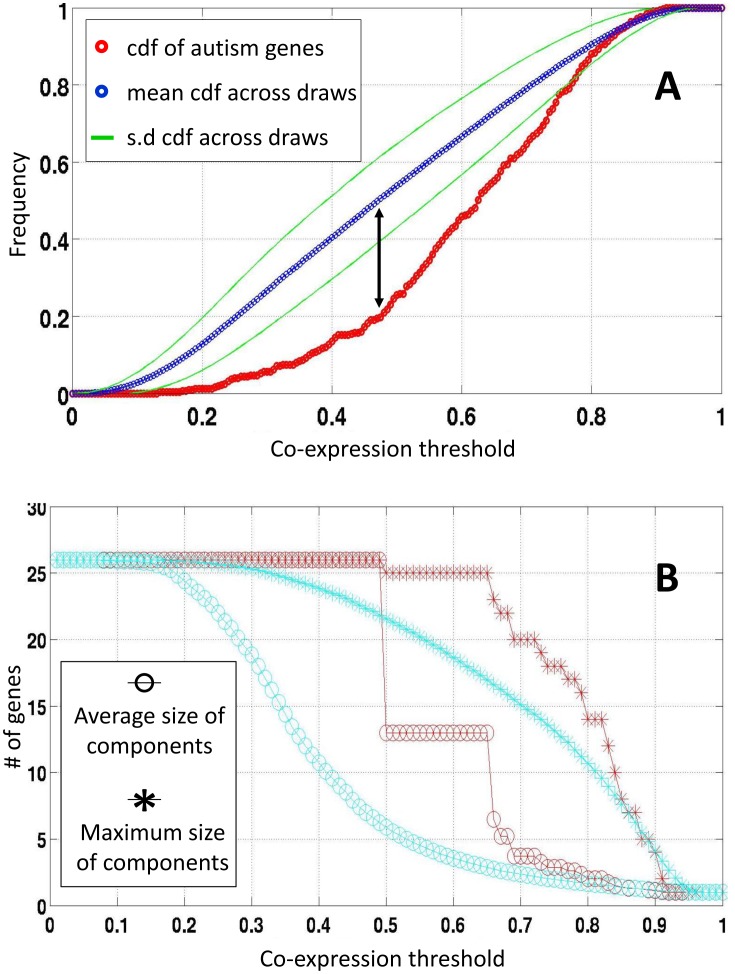
Co-expression characteristics of gene networks. Distributions of co-expression characteristics are depicted for the autism gene network (Red) and for the 3041 genes in the mouse Allen Brain Atlas database (Blue). (A) Cumulative distribution functions (CDFs). Black arrow indicates the maximal difference between the two CDFs. (B) Average and maximum sizes of connected components for different co-expression thresholds are plotted for the autism gene network (Red) and 1000 randomly generated gene networks of size G = 26 (Cyan).

Overall, 26 genes were found in the intersection of the autism-genes dataset and the dataset of high-quality expression genes from the Allen Brain Atlas (ABA) of the mouse brain (*AutRef84∩A_best_* = 26). These autism-related genes showed a higher degree of co-expression connectivity than all other genes in this dataset (Kolmogorov–Smirnov *P* = 5×10^−28^). Comparing the empirical distributions of co-expression values of the autism genes to the other genes in the Allen dataset revealed that the largest deviation between these distributions was at co-expression value of 47.53% (**Figure 1A**). Furthermore, we evaluated the connectivity of genes across different co-expression values. Here too, the average size of connected components (See methods) among autism gene was consistently larger than seen in 1000 randomly generated gene sets from the Allen database (**Figure 1B**).

**Figure 2:**
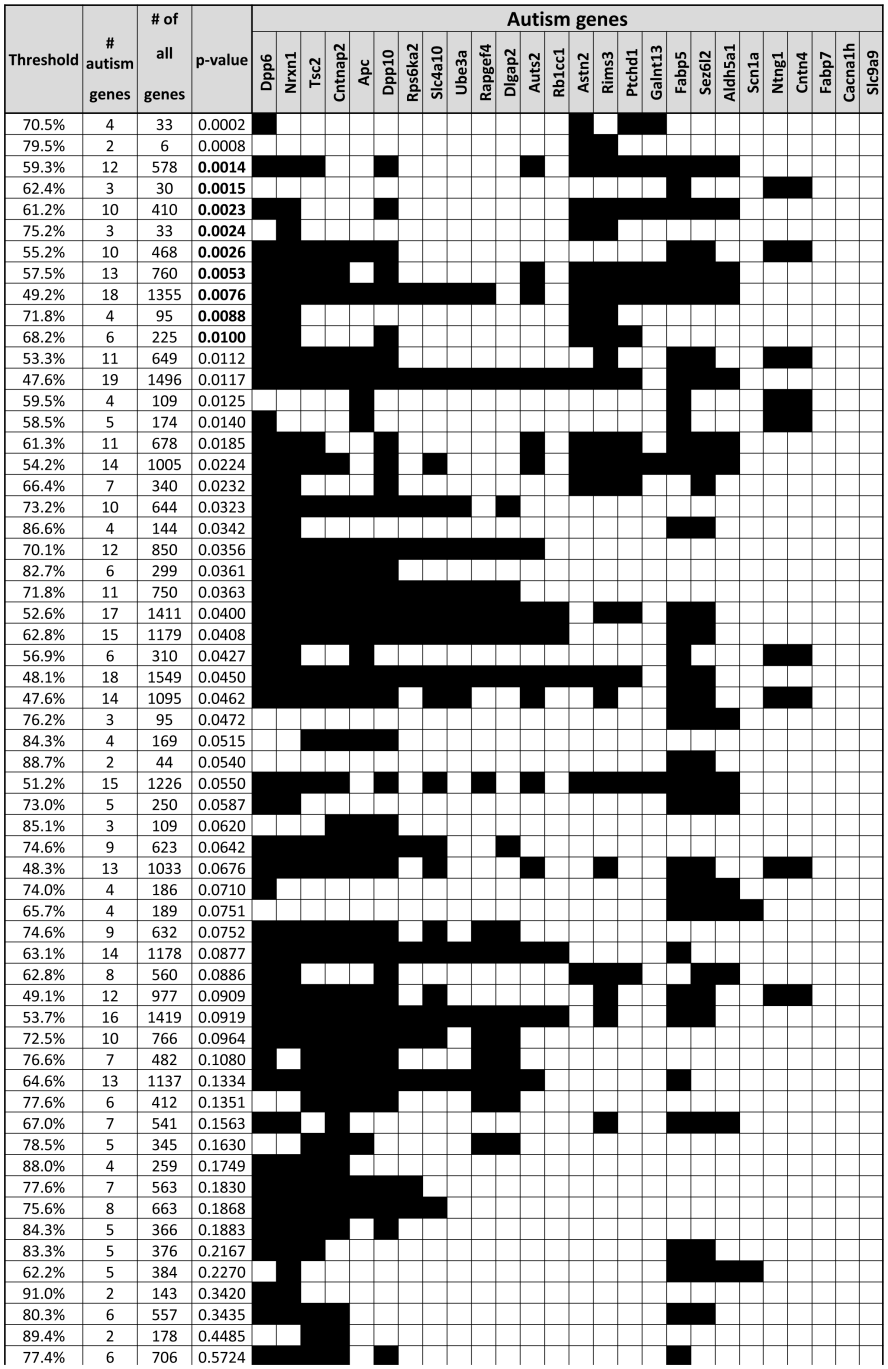
Cliques of co-expression genes delineated by co-expressed autism genes. The 59 cliques of co-expression genes containing ≥2 autism genes identified in our data are listed in the rows together with the minimal co-expression value in the clique, and numbers of autism genes and number of total genes in the clique. A p-value indicates the likelihood of finding this number of autism genes in the clique (based on 100,000 Monte Carlo simulations). The 26 autism genes included in the study are depicted in columns with black and white fillings indicating their presence and absence from a clique respectively.

**Figure 3:**
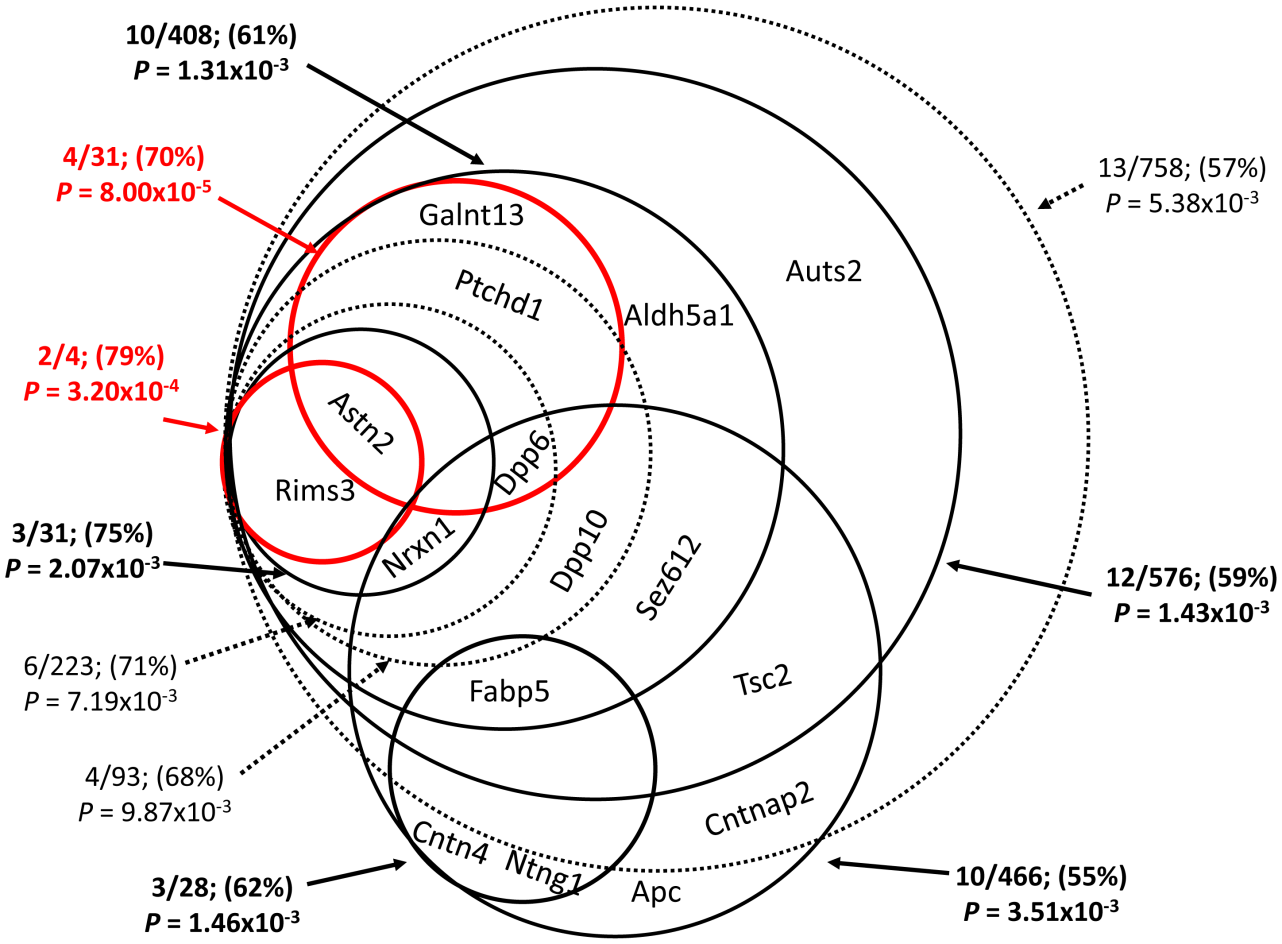
Cliques of co-expressed genes. Venn diagram for the ten cliques of co-expressed genes that are highly enriched with autism genes. For each clique, the following parameters are given: the number of autism genes/number of all genes in the clique, the co-expression threshold (in %) of the clique, and the p-value of the autism gene enrichment (using 100,000 Monte Carlo resampling procedure).

Next, we examined cliques (see methods) of co-expressed genes delineated by autism genes inter-connected at co-expression values of ≥47.53%. A total of 59 overlapping cliques were characterized containing on average 563.5 genes and 8.3 autism genes (**Figure 2**). Finally, using Monte Carlo simulations, we identified ten cliques that were significantly enriched with autism genes at *P*<0.01 (**Figure 3**). Of note, the two top ranked cliques remained significant (*P*<0.05) even after accounting for multiple testing using the conservative Bonferroni correction.

**Figure 4:**
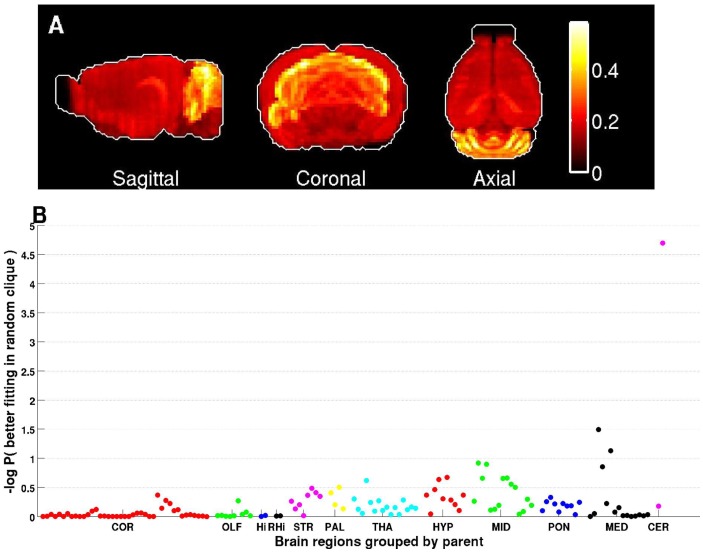
Expression and anatomical properties of Clique I. (A) Maximal-intensity projection of the sum of normalized expressions of genes in this clique highlight regions in the cerebellum. (B) The expression fittings in these regions are higher than expected by chance (P = 0.00002, based on 100,000 random permutations). The brain regions of the ABA at 200 micron resolutions (one dot per region on the figure) are grouped into the following main regions: COR (cerebral cortex), OLF (olfactory areas), Hi (hippocampal region), RHi (Retrohippocampal region), STR (striatum), PAL (pallidum), THA (thalamus), HYP (hypoyhalamus), MID,(midbrain), PON (pons), MED (medulla), CER (cerebellum).

**Figure 5:**
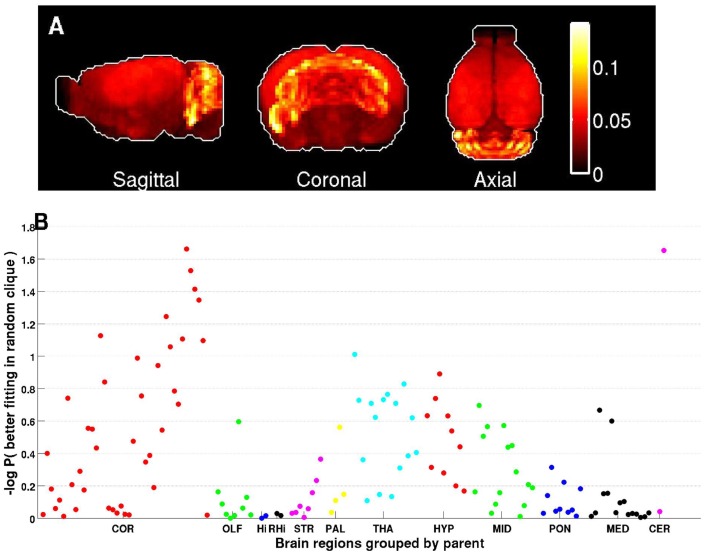
Expression and anatomical properties of Clique II. (A) Maximal-intensity projection of the sum of expressions of genes in this clique highlights the cerebellar cortex. (B) The fitting score in the cerebellar cortex is highest among all other brain regions and higher than expected by chance (*P* = 0.018, based on 100,000 random permutations). Some other regions in the cerebral cortex also show a slight deviation from expression values expected by chance (P<0.05). The brain regions of the ABA at 200 micron resolutions (one dot per region on the figure) are grouped into the following main regions: COR (cerebral cortex), OLF (olfactory areas), Hi (hippocampal region), RHi (Retrohippocampal region), STR (striatum), PAL (pallidum), THA (thalamus), HYP (hypoyhalamus), MID,(midbrain), PON (pons), MED (medulla), CER (cerebellum).

The top-ranked clique in our analysis (hereafter will be referred as Clique I), was delineated by the autism genes: *Ptchd1*, *Galnt13*, *Dpp6* and *Astn2*, and included another 29 genes, inter-connected with a co-expression values of ≥70% (**Supplementary Table S2**). The second top-ranked clique (hereafter will be referred as Clique II) included the autism genes: *Rims3*, and *Astn2*, and another four genes, all inter-connected at ≥79% co-expression level (**Supplementary Table S3**). Examining the neuroanatomical expression properties across a set of 134 brain regions of the left hemisphere (41 of which are cortical, and 93 subcortical) grouped by the 12 main brain regions according to the Allen Reference Atlas, revealed a significant over-expression of genes belonging to Cliques I in the cerebellar cortex (**Figures 4A, 4B** **, Supplementary Figure S1**). Genes belonging to Clique II also showed a slight over-expression in the cerebellar cortex (**Figures 5A, 5B**), as well as in several cortical regions, however these signals were much weaker than the one of clique I. Next, we asked if the over-expression in the cerebellum is a unique property of these two cliques. For that, we examined the neuroanatomical expression of all cliques in **Figure 2** and found only seven other cliques showing a similar over-expression in the cerebellar cortex. Interestingly, these cliques had substantial gene overlap with cliques I&II, and were ranked high in their autism gene enrichment scores (**Supplementary Table S4**), thus supporting the illumination of the cerebellar cortex in this analysis.

**Figure 6:**
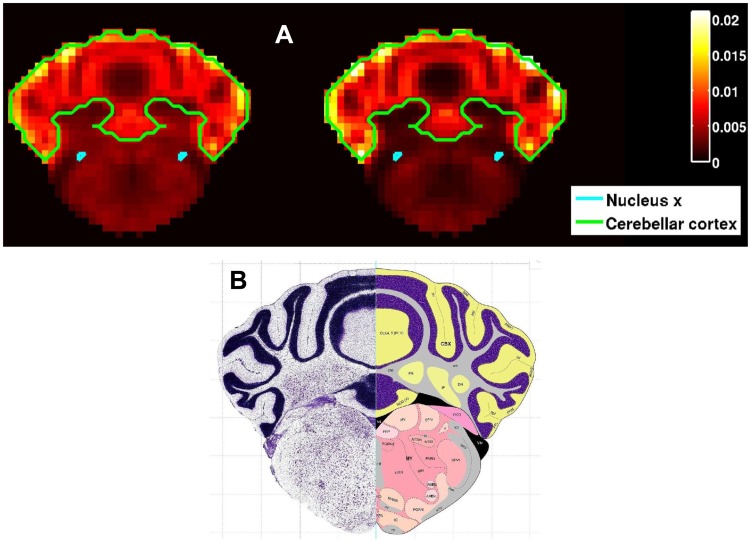
Cell type properties of Cliques I & II. (A) Sections of the sum of expressions of genes in cliques I (left) and II (right) are depicted through the most frontal coronal section of the cerebellum that intersects Nucleus X. The boundaries of the cerebellar cortex and of nucleus X are outlined. (B) The corresponding coronal section of the mouse brain in the Allen Mouse Brain Atlas, Allen Institute for Brain Science [57].

Finally, we asked if these two co-expressed cliques are associated with particular cell type or any other functional or cellular property. Examining cell-type-specific microarray data which we have for 64 cell types [43] revealed that 4 of them (stellate basket cells, granule cells, oligodendrocytes and Purkinje cells) are considerably populating the cerebellum (**Supplementary Figure S2**). Further, we looked through different coronal sections through the cerebellum from the Allen Reference Atlas [41] and visually compared them to sections of the sum of expressions of genes in Cliques I & II. **Figure 6** shows the normalized volumetric expression quantities of both cliques along with the closest coronal section of the mouse brain in the Allen Reference Atlas. One can see that the voxels with the most intense expression in both cliques tend to follow the granular layer. Hence, the results of these analyses suggest that genes in both clique I & II tend to be over-expressed in granule cells in the cerebellar cortex. Using the Bioconductor GOstats package in R, we two biological processes: “Transmission of nerve impulse” (*P* = 0.001842), and “Ion transport” (*P* = 0.000733) and one cellular component “Vesicles” (*P* = 0.001134) that were enriched with genes from Clique I (**Supplementary Table S5**). Unfortunately, the number of genes in Clique II was too small for this analysis.

## Discussion

In this study, we explored the co-expression network 26 autism genes within the framework of 3,041 genes exhibiting the highest-quality expression data in the Allen Mouse Brain Atlas database [30]–[33]. The significantly tighter co-expression connectivity among the 26 autism genes than other genes, implies common functional properties for these genes in the mouse brain. Further investigation into the co-expression patterns of these genes revealed two cliques of co-expressed genes that were significantly dominated by autism genes. Genes in both these cliques shown significant over-expression in the cerebellar cortex, and particularly in sections that are predominantly populated by granular cells. Some regions of the cerebral cortex are also highlighted by the second clique (**Figure 5**), but to a lesser extent than the cerebellar cortex. Another recent study of our group examining the expression of the same autism gene set (AutRef84) in different human tissues, found a statistically significant enrichment in the frontal cortex [44]. The cerebral cortex was highlighted in other neuroanatomical studies of autism in both human [45], [46] and mouse [47] and is known to play a central role in cognitive and emotional processing [48], which are key deficits in autism and other neuropsychiatric disorders. In addition, a recent neuroimaging study [49] highlighted functional sub-regions in the cerebellum as playing a role in both motor and cognitive tasks. Other genes associated with autism have been shown to play a role in the development of this region [50]–[53]. Our results, provide additional support in the potential involvement of the cerebellum in autism etiology, and suggest additional candidate genes that are also over-expressed in the cerebellar cortex.

Two recent transcriptomic analyses in human brains [6], [54] revealed additional co-expression modules enriched with autism-associated genes. Some of these modules partially overlap with our findings in either gene content or brain regions, suggesting common functional and neuroanatomical properties of autism gene in both human and mouse brains. Together, these studies provide new insights into the specific gene networks and brain regions that could be involved in autism etiology.

A major strength of our study is the utilization of the Allen Mouse Brain Atlas [30]–[33] which comprises a high-resolution genome-wide exploration of gene expression in the adult mouse brain. This data allows one to explore gene expression properties up to a resolution of 200 microns, which provide a good distinction between different brain regions as well as potentially tell apart different sub-regions and cell types. Another advantage of this study is the focus on those genes exhibiting the highest expression correlation between the coronal and sagittal sections [35] as well as restricting the autism gene to a subset demonstrating, to the best of our knowledge, the most compelling associations to autism susceptibility. These strict criteria reduce the chances of erroneous results. Our study has also some pitfalls. First, the analyses were done on data from mouse brains. Since autism is a human condition, one may ask how well finding of this study apply to human brain. A recent study comparing postsynaptic protein composition between mouse and human suggest a high correlation between these two mammals in those matters [55]. Nevertheless, similar analyses in the human brain are still required to provide a finer validity to our findings. In addition, the strict criteria used here, restricted the number of studied genes to 3, 041 and 26 autism genes which are roughly represent 15% and 31% of the genes in the Allen Brain Atlas and AutDB datasets respectively. Such a small number of genes might results in false negatives and hence might miss other co-expression properties and brain regions associated with autism. Hence, larger studies are needed to complement the results of our analysis.

In conclusions, our study provides unique insights into the neuroanatomical co-expression properties of genes associated with autism in the mouse brain and suggest specific regions implicated in autism etiology. Complementing these findings with additional genomics and neuroimaging analyses from both mouse and human brains would help gaining a broader picture of the autistic brain.

## Supporting Information

Figure S1 **ISH images of the autism genes of Clique I.** Imaged sections of ISH-treated brains (close to bregma), for (A) Astn2, (B) Galnt13, and (C) Ptchd1 with the cerebellar cortex clearly visible.
(TIF)

Figure S2 **Estimated brain-wide density for (A) Stellate Basket Cells, (B) Granule Cells, and (C) Mature Oligodendrocytes.** These data correspond to microarray data from [56], using the data sets estimated in [43] to have the highest purity. Interestingly, the estimated brain-wide densities are almost zero in the cerebral cortex, suggesting that cell types characterized by their transcriptomes are indeed specific to the cerebellar cortex.
(TIF)

Table S1 **AutRef84: A Reference set of** ***Rare*** **and** ***Syndromic*** **ASD-linked genes.**
(XLSX)

Table S2 **Co-expression of genes in Clique I.** The pairwise co-expression values between all 33 genes in Clique I are depicted and color-coded. The four genes already associated with autism are highlighted in yellow.
(XLSX)

Table S3 **Co-expression of genes in Clique II.** The pairwise co-expression values between all six genes in Clique II are depicted and color-coded. The two genes already associated with autism are highlighted in yellow.
(XLSX)

Table S4 **Overlap of cliques displaying overexpression in the cerebellum with cliques I & II.**
(DOC)

Table S5 **Results of the Bioconductor GOstat analysis for genes belonging to Clique I.**
(DOC)

## References

[1] Levy SE, Mandell DS, Schultz RT (2009) Autism. Lancet 374: 1627–1638. 19819542 10.1016/S0140-6736(09)61376-3 PMC2863325

[2] Lord C (2011) Epidemiology: How common is autism? Nature 474: 166–168. 21654793 10.1038/474166a

[3] Newschaffer CJ, Croen LA, Daniels J, Giarelli E, Grether JK, et al (2007) The epidemiology of autism spectrum disorders. Annu Rev Public Health 28: 235–258. 17367287 10.1146/annurev.publhealth.28.021406.144007

[4] Walsh CA, Morrow EM, Rubenstein JL (2008) Autism and brain development. Cell 135: 396–400. 18984148 10.1016/j.cell.2008.10.015 PMC2701104

[5] Amaral DG, Schumann CM, Nordahl CW (2008) Neuroanatomy of autism. Trends Neurosci 31: 137–145. 18258309 10.1016/j.tins.2007.12.005

[6] Voineagu I, Wang X, Johnston P, Lowe JK, Tian Y, et al (2011) Transcriptomic analysis of autistic brain reveals convergent molecular pathology. Nature 474: 380–384. 21614001 10.1038/nature10110 PMC3607626

[7] Carper RA, Courchesne E (2005) Localized enlargement of the frontal cortex in early autism. Biological Psychiatry 57: 126–133. 15652870 10.1016/j.biopsych.2004.11.005

[8] Courchesne E, Pierce K (2005) Why the frontal cortex in autism might be talking only to itself: local over-connectivity but long-distance disconnection. Current Opinion in Neurobiology 15: 225–230. 15831407 10.1016/j.conb.2005.03.001

[9] Etherton M, Foldy C, Sharma M, Tabuchi K, Liu X, et al (2011) Autism-linked neuroligin-3 R451C mutation differentially alters hippocampal and cortical synaptic function. Proc Natl Acad Sci U S A 108: 13764–13769. 21808020 10.1073/pnas.1111093108 PMC3158170

[10] Herbert MR (2011) SHANK3, the synapse, and autism. N Engl J Med 365: 173–175. 21751912 10.1056/NEJMcibr1104261

[11] Gilman SR, Iossifov I, Levy D, Ronemus M, Wigler M, et al (2011) Rare de novo variants associated with autism implicate a large functional network of genes involved in formation and function of synapses. Neuron 70: 898–907. 21658583 10.1016/j.neuron.2011.05.021 PMC3607702

[12] Garber K (2007) Neuroscience - Autism's cause may reside in abnormalities at the synapse. Science 317: 190–191. 17626859 10.1126/science.317.5835.190

[13] Bourgeron T (2009) A synaptic trek to autism. Current Opinion in Neurobiology 19: 231–234. 19545994 10.1016/j.conb.2009.06.003

[14] Durand CM, Betancur C, Boeckers TM, Bockmann J, Chaste P, et al (2007) Mutations in the gene encoding the synaptic scaffolding protein SHANK3 are associated with autism spectrum disorders. Nat Genet 39: 25–27. 17173049 10.1038/ng1933 PMC2082049

[15] Szatmari P, Paterson AD, Zwaigenbaum L, Roberts W, Brian J, et al (2007) Mapping autism risk loci using genetic linkage and chromosomal rearrangements. Nat Genet 39: 319–328. 17322880 10.1038/ng1985 PMC4867008

[16] Jacquemont ML, Sanlaville D, Redon R, Raoul O, Cormier-Daire V, et al (2006) Array-based comparative genomic hybridisation identifies high frequency of cryptic chromosomal rearrangements in patients with syndromic autism spectrum disorders. J Med Genet 43: 843–849. 16840569 10.1136/jmg.2006.043166 PMC2563185

[17] Cook EH Jr, Scherer SW (2008) Copy-number variations associated with neuropsychiatric conditions. Nature 455: 919–923. 18923514 10.1038/nature07458

[18] Levy D, Ronemus M, Yamrom B, Lee YH, Leotta A, et al (2011) Rare de novo and transmitted copy-number variation in autistic spectrum disorders. Neuron 70: 886–897. 21658582 10.1016/j.neuron.2011.05.015

[19] Anney R, Klei L, Pinto D, Regan R, Conroy J, et al (2010) A genome-wide scan for common alleles affecting risk for autism. Hum Mol Genet 19: 4072–4082. 20663923 10.1093/hmg/ddq307 PMC2947401

[20] Myers RA, Casals F, Gauthier J, Hamdan FF, Keebler J, et al (2011) A population genetic approach to mapping neurological disorder genes using deep resequencing. PLoS Genet 7: e1001318. 21383861 10.1371/journal.pgen.1001318 PMC3044677

[21] Iossifov I, Ronemus M, Levy D, Wang Z, Hakker I, et al (2012) De novo gene disruptions in children on the autistic spectrum. Neuron 74: 285–299. 22542183 10.1016/j.neuron.2012.04.009 PMC3619976

[22] Neale BM, Kou Y, Liu L, Ma'ayan A, Samocha KE, et al (2012) Patterns and rates of exonic de novo mutations in autism spectrum disorders. Nature 485: 242–245. 22495311 10.1038/nature11011 PMC3613847

[23] O'Roak BJ, Vives L, Girirajan S, Karakoc E, Krumm N, et al (2012) Sporadic autism exomes reveal a highly interconnected protein network of de novo mutations. Nature 485: 246–250. 22495309 10.1038/nature10989 PMC3350576

[24] Sanders SJ, Murtha MT, Gupta AR, Murdoch JD, Raubeson MJ, et al (2012) De novo mutations revealed by whole-exome sequencing are strongly associated with autism. Nature 485: 237–241. 22495306 10.1038/nature10945 PMC3667984

[25] Basu SN, Kollu R, Banerjee-Basu S (2009) AutDB: a gene reference resource for autism research. Nucleic Acids Res 37: D832–836. 19015121 10.1093/nar/gkn835 PMC2686502

[26] Moy SS, Nadler JJ, Magnuson TR, Crawley JN (2006) Mouse models of autism spectrum disorders: the challenge for behavioral genetics. Am J Med Genet C Semin Med Genet 142C: 40–51. 16419099 10.1002/ajmg.c.30081

[27] Buxbaum JD, Betancur C, Bozdagi O, Dorr NP, Elder GA, et al (2012) Optimizing the phenotyping of rodent ASD models: Enrichment analysis of mouse and human neurobiological phenotypes associated with high-risk autism genes identifies morphological, electrophysiological, neurological, and behavioral features. Mol Autism 3: 1. 22348382 10.1186/2040-2392-3-1 PMC3337792

[28] Moy SS, Nadler JJ (2008) Advances in behavioral genetics: mouse models of autism. Mol Psychiatry 13: 4–26. 17848915 10.1038/sj.mp.4002082

[29] Kumar A, Wadhawan R, Swanwick CC, Kollu R, Basu SN, et al (2011) Animal model integration to AutDB, a genetic database for autism. BMC Med Genomics 4: 15. 21272355 10.1186/1755-8794-4-15 PMC3042898

[30] Hawrylycz M, Baldock RA, Burger A, Hashikawa T, Johnson GA, et al (2011) Digital atlasing and standardization in the mouse brain. PLoS Comput Biol 7: e1001065. 21304938 10.1371/journal.pcbi.1001065 PMC3033370

[31] Hawrylycz M, Ng L, Page D, Morris J, Lau C, et al (2011) Multi-scale correlation structure of gene expression in the brain. Neural Netw 24: 933–942. 21764550 10.1016/j.neunet.2011.06.012

[32] Lein ES, Hawrylycz MJ, Ao N, Ayres M, Bensinger A, et al (2007) Genome-wide atlas of gene expression in the adult mouse brain. Nature 445: 168–176. 17151600 10.1038/nature05453

[33] Ng L, Bernard A, Lau C, Overly CC, Dong HW, et al (2009) An anatomic gene expression atlas of the adult mouse brain. Nat Neurosci 12: 356–362. 19219037 10.1038/nn.2281

[34] AutDB MindSpec.

[35] Bohland JW, Bokil H, Pathak SD, Lee CK, Ng L, et al (2010) Clustering of spatial gene expression patterns in the mouse brain and comparison with classical neuroanatomy. Methods 50: 105–112. 19733241 10.1016/j.ymeth.2009.09.001

[36] Grange P, Bohland JW, Hawrylycz M, Mitra PP (2012) arXiv:1211.6177 [q-bio.NC]. Brain Gene Expression Analysis: a MATLAB toolbox for the analysis of brain-wide gene-expression data. Cornell University Library

[37] P G, M H, Mitra P (2013) Computational neuroanatomy and co-expression of genes in the adult mouse brain, analysis tools for the Allen Brain Atlas. Quantitative Biology 1: 91–100.

[38] Luce RD, Perry AD (1949) A method of matrix analysis of group structure. Psychometrika 14: 95–116. 18152948 10.1007/BF02289146

[39] Tanay A, Sharan R, Shamir R (2002) Discovering statistically significant biclusters in gene expression data. Bioinformatics 18 Suppl 1: S136–144. 12169541 10.1093/bioinformatics/18.suppl_1.s136

[40] Grange P, Mitra PP (2012) Computational neuroanatomy and gene expression: Optimal sets of marker genes for brain regions. IEEE Conference on Information Sciences and Systems. Princeton.

[41] Dong HW, editor (2008) The Allen Reference Atlas: a digital brain atlas of the C57BL/6J male mouse. John Wiley & Sons, Inc.

[42] Gentleman RC, Carey VJ, Bates DM, Bolstad B, Dettling M, et al (2004) Bioconductor: open software development for computational biology and bioinformatics. Genome Biol 5: R80. 15461798 10.1186/gb-2004-5-10-r80 PMC545600

[43] Okaty BW, Sugino K, Nelson SB (2011) A quantitative comparison of cell-type-specific microarray gene expression profiling methods in the mouse brain. PLoS One 6: e16493. 21304595 10.1371/journal.pone.0016493 PMC3029380

[44] Kumar A, Swanwick CC, Johnson N, Menashe I, Basu SN, et al (2011) A Brain Region-Specific Predictive Gene Map for Autism Derived by Profiling a Reference Gene Set. PLoS One 6: e28431. 22174805 10.1371/journal.pone.0028431 PMC3235126

[45] Tetreault NA, Hakeem AY, Jiang S, Williams BA, Allman E, et al (2012) Microglia in the cerebral cortex in autism. J Autism Dev Disord 42: 2569–2584. 22466688 10.1007/s10803-012-1513-0

[46] Avino TA, Hutsler JJ (2010) Abnormal cell patterning at the cortical gray-white matter boundary in autism spectrum disorders. Brain Res 1360: 138–146. 20816758 10.1016/j.brainres.2010.08.091

[47] Kumar M, Kim S, Pickup S, Chen R, Fairless AH, et al (2012) Longitudinal in-vivo diffusion tensor imaging for assessing brain developmental changes in BALB/cJ mice, a model of reduced sociability relevant to autism. Brain Res 1455: 56–67. 22513103 10.1016/j.brainres.2012.03.041 PMC3340503

[48] Rubenstein JL (2011) Annual Research Review: Development of the cerebral cortex: implications for neurodevelopmental disorders. J Child Psychol Psychiatry 52: 339–355. 20735793 10.1111/j.1469-7610.2010.02307.x PMC3429600

[49] Stoodley CJ, Valera EM, Schmahmann JD (2012) Functional topography of the cerebellum for motor and cognitive tasks: an fMRI study. Neuroimage 59: 1560–1570. 21907811 10.1016/j.neuroimage.2011.08.065 PMC3230671

[50] Kwan KY, Lam MM, Johnson MB, Dube U, Shim S, et al (2012) Species-dependent posttranscriptional regulation of NOS1 by FMRP in the developing cerebral cortex. Cell 149: 899–911. 22579290 10.1016/j.cell.2012.02.060 PMC3351852

[51] Wegiel J, Schanen NC, Cook EH, Sigman M, Brown WT, et al (2012) Differences between the pattern of developmental abnormalities in autism associated with duplications 15q11.2–q13 and idiopathic autism. J Neuropathol Exp Neurol 71: 382–397. 22487857 10.1097/NEN.0b013e318251f537 PMC3612833

[52] Eagleson KL, Campbell DB, Thompson BL, Bergman MY, Levitt P (2011) The autism risk genes MET and PLAUR differentially impact cortical development. Autism Res 4: 68–83. 21328570 10.1002/aur.172 PMC3644181

[53] Hedrick A, Lee Y, Wallace GL, Greenstein D, Clasen L, et al (2012) Autism risk gene MET variation and cortical thickness in typically developing children and adolescents. Autism Res 5: 434–439. 23097380 10.1002/aur.1256 PMC3528800

[54] Ben-David E, Shifman S (2012) Networks of neuronal genes affected by common and rare variants in autism spectrum disorders. PLoS Genet 8: e1002556. 22412387 10.1371/journal.pgen.1002556 PMC3297570

[55] Bayes A, Collins MO, Croning MD, van de Lagemaat LN, Choudhary JS, et al (2012) Comparative study of human and mouse postsynaptic proteomes finds high compositional conservation and abundance differences for key synaptic proteins. PLoS One 7: e46683. 23071613 10.1371/journal.pone.0046683 PMC3465276

[56] Doyle JP, Dougherty JD, Heiman M, Schmidt EF, Stevens TR, et al (2008) Application of a translational profiling approach for the comparative analysis of CNS cell types. Cell 135: 749–762. 19013282 10.1016/j.cell.2008.10.029 PMC2763427

[57] Allen Institute for Brain Science, Allen Mouse Brain Atlas [Internet]. Available from: http://mouse.brain-map.org/

