# Supplementary figures and images for: Co-expression Profiling of Autism Genes in the Mouse Brain
Source: PLoS Comput Biol. 2013 Jul 25;9(7):e1003128. doi: 10.1371/journal.pcbi.1003128 (PMC3723491; doi:10.1371/journal.pcbi.1003128)

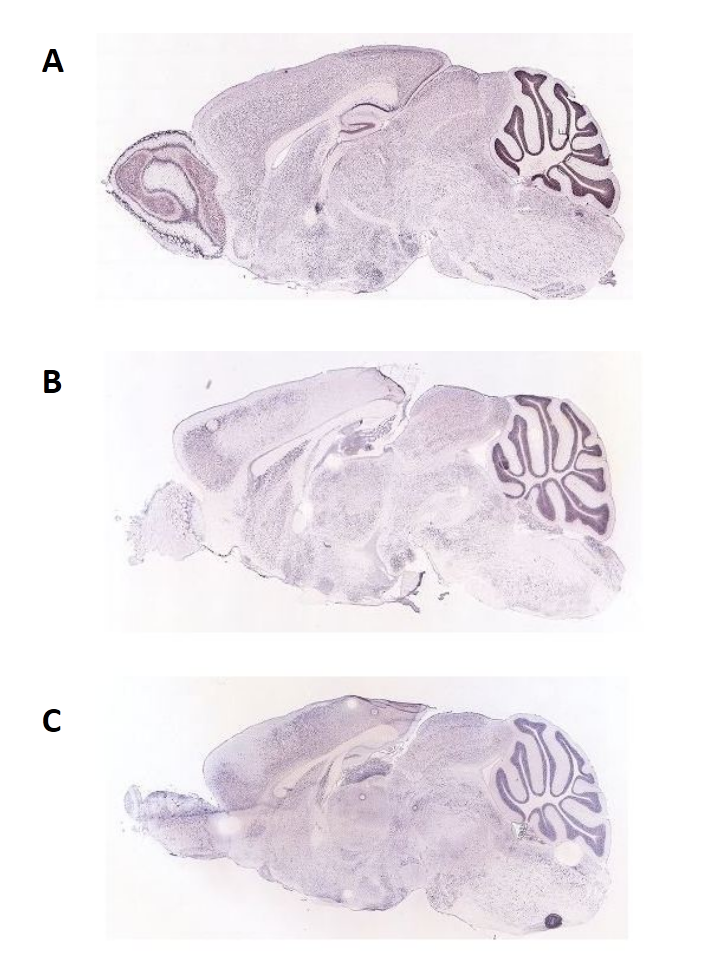

Supplement: Figure S1 — ISH images of the autism genes of Clique I. Imaged sections of ISH-treated brains (close to bregma), for (A) Astn2, (B) Galnt13, and (C) Ptchd1 with the cerebellar cortex clearly visible. (TIF) [file pcbi.1003128.s001.tif]

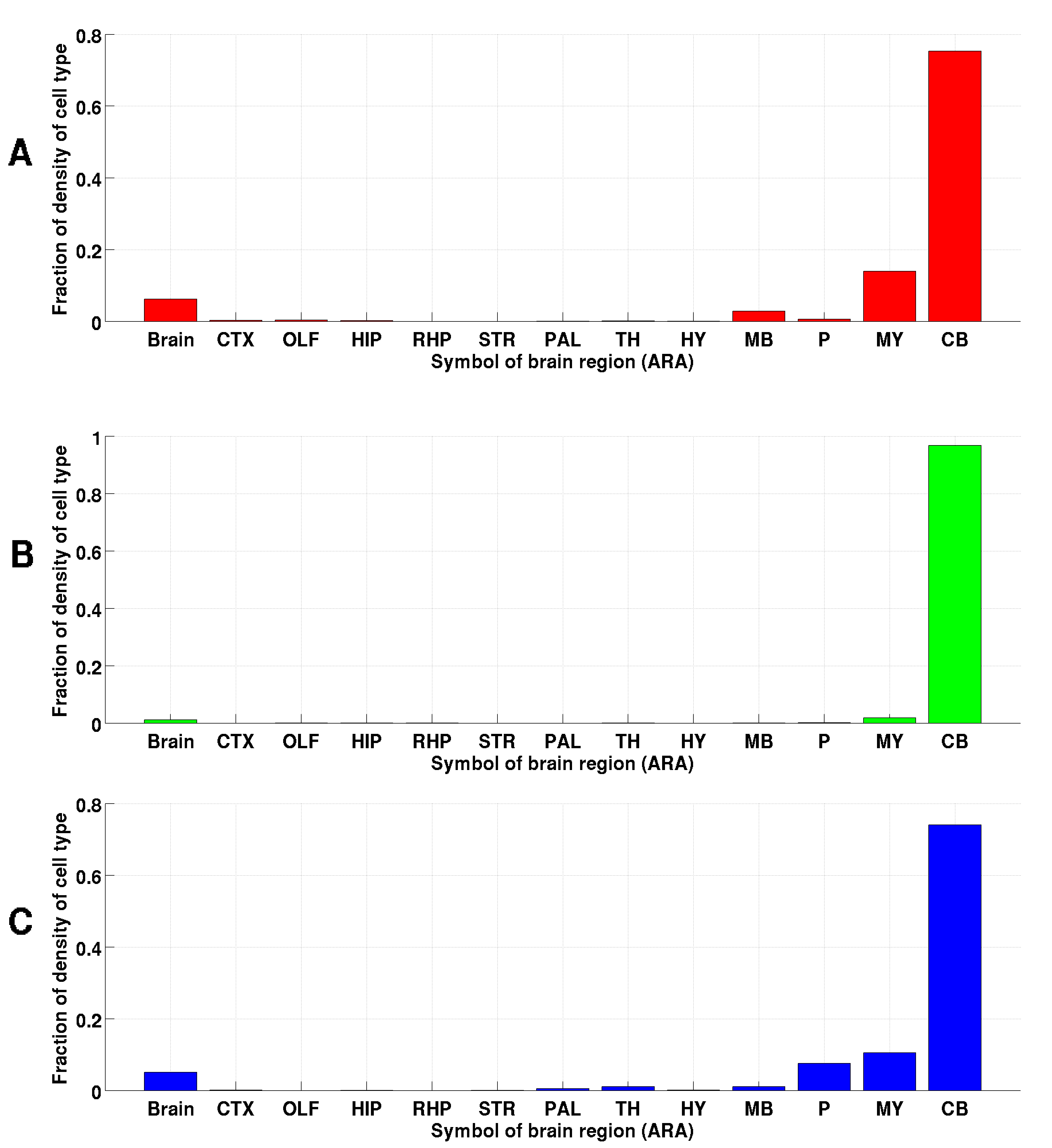

Supplement: Figure S2 — Estimated brain-wide density for (A) Stellate Basket Cells, (B) Granule Cells, and (C) Mature Oligodendrocytes. These data correspond to microarray data from [56], using the data sets estimated in [43] to have the highest purity. Interestingly, the estimated brain-wide densities are almost zero in the cerebral cortex, suggesting that cell types characterized by their transcriptomes are indeed specific to the cerebellar cortex. (TIF) [file pcbi.1003128.s002.tif]
