# Supplementary material for: Co-expression Profiling of Autism Genes in the Mouse Brain
Source: PLoS Comput Biol. 2013 Jul 25;9(7):e1003128. doi: 10.1371/journal.pcbi.1003128 (PMC3723491; doi:10.1371/journal.pcbi.1003128)
Supplement: Table S4 — Overlap of cliques displaying overexpression in the cerebellum with cliques I & II. (DOC) [file pcbi.1003128.s006.doc]

**Supplementary Table S4:** Overlap of cliques displaying overexpression in the cerebellum with cliques I & II

| **Index of clique in Table 1** | **Number of autism genes** | **Number of Atlas genes** | **Number of autism genes in Clique I** | **Number of autism genes in Clique II** | **Number of Atlas genes also found in Clique I** | **Number of Atlas genes also found in Clique II** |
| --- | --- | --- | --- | --- | --- | --- |
| 3 | 12 | 532 | 4 | 2 | 32 | 6 |
| 5 | 10 | 410 | 4 | 2 | 32 | 6 |
| 6 | 3 | 33 | 1 (*Astn2*) | 2 | 14 | 6 |
| 8 | 13 | 760 | 4 | 2 | 33 | 6 |
| 10 | *4* | 95 | 2 *(Astn2, Dpp6)* | 2 | 26 | 6 |
| 11 | 6 | 225 | 3 *(Astn2, Dpp6, Ptchd1)* | 2 | 31 | 6 |
| 18 | 7 | 340 | 3 *(Astn2, Dpp6, Ptchd1)* | 2 | 31 | 6 |
