# Supplementary material for: Co-expression Profiling of Autism Genes in the Mouse Brain
Source: PLoS Comput Biol. 2013 Jul 25;9(7):e1003128. doi: 10.1371/journal.pcbi.1003128 (PMC3723491; doi:10.1371/journal.pcbi.1003128)
Supplement: Table S5 — Results of the Bioconductor GOstat analysis for genes belonging to Clique I. (DOC) [file pcbi.1003128.s007.doc]

**Supplementary Table S5:** Results of the Bioconductor GOstat analysis for genes belonging to Clique I

| **GO Category** | **GO term** | **Cut off** | **P-value** | **No. of genes** | **Genes** |
| --- | --- | --- | --- | --- | --- |
| Biological process | regulation of cellular catabolic process | 2X | 0.0475 | 3 | SOCS5, S1PR1, CNR1 |
|  | cytokine-mediated signaling pathway |  | 0.027676 | 3 | SOCS5, CAMK2D, DARC |
|  | generation of a signal involved in cell-cell signaling |  | 0.022074 | 3 | SLC6A1, SYT2, CNR1 |
|  | cellular homeostasis |  | 0.034879 | 4 | DPP6, CAMK2D, S1PR1, CNR1 |
|  | cellular ion homeostasis |  | 0.018297 | 4 | DPP6, CAMK2D, S1PR1, CNR1 |
|  | regulation of system process |  | 0.00792 | 4 | ATP1A1, CAMK2D, SLC6A1, CNR1 |
|  | actin cytoskeleton organization |  | 0.006292 | 4 | S1PR1, MTSS1, DAAM2, CORO2B |
|  | monovalent inorganic cation transport |  | 0.00155 | 4 | ATP1A1, DPP6, SLC12A3, KCNC1 |
|  | system process |  | 0.041776 | 5 | DPP6, S1PR1, SYT2 , KCNC1, PTCHD1 |
|  | lipid metabolic process |  | 0.048759 | 5 | ATP1A1, PLTP, PLA2G7, C14orf1, CNR1 |
|  | regulation of transport |  | 0.029259 | 5 | ATP1A1, DPP6, CAMK2D, SLC6A1, CNR1 |
|  | transmission of nerve impulse |  | 0.001842 | 5 | DPP6, S1PR1, SYT2, KCNC1, CAMK2D |
|  | ion transport |  | 0.000733 | 7 | ATP1A1, DPP6, CA4, CAMK2D, SLC12A3, CNR1, KCNC1 |
|  | cytokine-mediated signaling pathway | Median | 0.027676 | 3 | SOCS5, CAMK2D, DARC |
|  | generation of a signal involved in cell-cell signaling |  | 0.022074 | 3 | SLC6A1, SYT2, CNR1 |
|  | cellular ion homeostasis |  | 0.018297 | 4 | DPP6, CAMK2D, S1PR1, CNR1 |
|  | regulation of system process |  | 0.00792 | 4 | ATP1A1, CAMK2D, SLC6A1, CNR1 |
|  | actin cytoskeleton organization |  | 0.006292 | 4 | S1PR1, MTSS1, DAAM2, CORO2B |
|  | monovalent inorganic cation transport |  | 0.00155 | 4 | ATP1A1, DPP6, SLC12A3, KCNC1 |
|  | transmission of nerve impulse |  | 0.001842 | 5 | DPP6, S1PR1, SYT2, KCNC1, CAMK2D |
|  | ion transport |  | 0.000733 | 7 | ATP1A1, DPP6, CA4, CAMK2D, SLC12A3, CNR1, KCNC1 |
| Cellular component | cytoplasmic membrane-bounded vesicle | 2X | 0.041902 | 3 | ATP1A1, CAMK2D, MTSS1 |
|  | cytoplasmic vesicle part |  | 0.03219 | 3 | CA4, CAMK2D, SYT2 |
|  | vesicle membrane |  | 0.024074 | 3 | CA4, CAMK2D, SYT2 |
|  | secretory granule |  | 0.009452 | 3 | CHGB, CA4, SYT2 |
|  | sarcolemma |  | 0.000584 | 3 | ATP1A1, CA4, CAMK2D |
|  | intrinsic to plasma membrane |  | 0.006199 | 7 | ATP1A1, CA4, SLC6A1, SLC12A3, CNR1, MPP3, KCNC1 |
|  | vesicle |  | 0.001134 | 7 | ATP1A1, CHGB, CA4, CAMK2D, MTSS1,SYT2, C14orf1 |
|  | cytoplasmic vesicle part | Median | 0.03219 | 3 | CA4, CAMK2D, SYT2 |
|  | vesicle membrane |  | 0.024074 | 3 | CA4, CAMK2D, SYT2 |
|  | secretory granule |  | 0.009452 | 3 | CHGB, CA4, SYT2 |
|  | sarcolemma |  | 0.000584 | 3 | ATP1A1, CA4, CAMK2D |
|  | vesicle |  | 0.001134 | 7 | ATP1A1, CHGB, CA4, CAMK2D, MTSS1,SYT2, C14orf1 |
| Molecular Function | inorganic cation transmembrane transporter activity | 2X | 0.049636 | 3 | ATP1A1, SLC6A1, KCNC1 |
|  | actin binding |  | 0.022207 | 3 | SYNE1 , DAAM2, CORO2B |
|  | active transmembrane transporter activity |  | 0.024112 | 3 | ATP1A1, SLC6A1, SLC12A3 |
|  | ion transmembrane transporter activity |  | 0.039485 | 4 | ATP1A1, SLC6A1, SLC12A3, KCNC1 |
|  | signaling receptor activity |  | 0.033413 | 5 | S1PR1, GPR37L1, DARC, CNR1, PTCHD1 |
|  | inorganic cation transmembrane transporter activity | Median | 0.049636 | 3 | ATP1A1, SLC6A1, KCNC1 |
|  | actin binding |  | 0.022207 | 3 | SYNE1 , DAAM2, CORO2B |
|  | active transmembrane transporter activity |  | 0.024112 | 3 | ATP1A1, SLC6A1, SLC12A3 |
|  |  |  |  |  |  |
